# Asynchronous recovery of predators and prey conditions resilience to drought in a neotropical ecosystem

**DOI:** 10.1038/s41598-022-12537-2

**Published:** 2022-05-19

**Authors:** Thomas Ruiz, Jean-François Carrias, Camille Bonhomme, Vinicius F. Farjalla, Vincent E. J. Jassey, Joséphine Leflaive, Arthur Compin, Céline Leroy, Bruno Corbara, Diane S. Srivastava, Régis Céréghino

**Affiliations:** 1 Laboratoire Microorganismes, Génome Et Environnement, CNRS, Université Clermont Auvergne, Clermont-Ferrand, France; 2 Departamento de Ecología, Instituto de Biologia, Universidade Federal Do Rio de Janeiro (UFRJ), Ilha Do Fundão, Rio de Janeiro, Brazil; 3 AMAP, Université de Montpellier, CIRAD, CNRS, INRAE, IRD, Montpellier, France; 4 Laboratoire Écologie Fonctionnelle Et Environnement, Université de Toulouse, CNRS, Toulouse INP, Université Toulouse 3—Paul Sabatier (UT3), Toulouse, France; 5 ECOFOG, CNRS, CIRAD, INRAE, Université Des Antilles, Université de Guyane, Kourou, France; 6 Department of Zoology & Biodiversity Research Centre, University of British Columbia, Vancouver, Canada

**Keywords:** Ecology, Ecosystem ecology, Freshwater ecology, Tropical ecology

## Abstract

The predicted increase in the intensity and frequency of drought events associated with global climate change will impose severe hydrological stress to freshwater ecosystems, potentially altering their structure and function. Unlike freshwater communities’ direct response to drought, their post-drought recovery capacities remain understudied despite being an essential component driving ecosystem resilience. Here we used tank bromeliad as model ecosystem to emulate droughts of different duration and then assess the recovery capacities of ecosystem structure and function. We followed macroinvertebrate predator and prey biomass to characterize the recovery dynamics of trophic structure (i.e. predator–prey biomass ratio) during the post-drought rewetting phase. We showed that drought significantly affects the trophic structure of macroinvertebrates by reducing the predator–prey biomass ratio. The asynchronous recovery of predator and prey biomass appeared as a critical driver of the post-drought recovery trajectory of trophic structure. Litter decomposition rate, which is an essential ecosystem function, remained stable after drought events, indicating the presence of compensatory effects between detritivores biomass and detritivores feeding activity. We conclude that, in a context of global change, the asynchrony in post-drought recovery of different trophic levels may impact the overall drought resilience of small freshwater ecosystems in a more complex way than expected.

## Introduction

The frequency and intensity of droughts have increased over recent decades, affecting both natural ecosystems and human well-being^1^. Such droughts can have adverse consequences on ecosystems and agroecosystems, upon which humans ultimately depend^2–4^. Whilst all ecosystems types are vulnerable to drought^5–7^, alterations of hydrological cycles and water deficit are major threats to freshwater ecosystem structure and functioning^8^. Small waterbodies (i.e. headwater streams, ponds, ditches, etc.) are particularly sensitive to hydrological stress. Short dry periods can lead to the concentration of nutrients and organisms in small water volumes, with consequences on biotic interactions^9^, and can even entirely dry out the system^6,10^. Although small waterbodies are the most numerous freshwater ecosystems globally^11,12^, account for a substantial fraction of freshwater biodiversity^13^, and provide many ecosystem services^11^, they are among the least studied ecosystems^14^. Therefore, the predicted increase in the frequency and intensity of droughts over the twenty-first century (IPCC 2014) questions the long-term integrity of these ecosystems worldwide, urging scientists to explore the mechanisms that underlie community and ecosystem resilience to drought in small waterbodies.

Predicting the response of small waterbodies to drought intensification requires the integration of two principal processes^15^: the immediate resistance of community and ecosystem processes to drought, and their resilience after drought. The resistance of processes directly reflects the ability of resident communities to survive during dry periods in active and/or dormant stages^16,17^. This resistance depends on community composition since tolerance to drought can be highly variable from one species to another^18,19^. Some studies have assessed the drought resistance of freshwater species^20^ or communities^21,22^, but few of them focused specifically on small waterbodies^23^. While these studies documented the immediate outcomes of drought on ecosystem structure and functions using before-after comparisons^24^ they generally ignored the capacities of communities to recover during the post-drought, rewetting phase. Yet, the recovery capacity, and especially recovery time (i.e., the time required to return to a pre-disturbance state), is critical for community structure and ecosystem function. Indeed, if a new drought occurs before the full recovery of the system, the ecosystem may shift toward a new equilibrium^25^. Therefore, current gaps in our knowledge of post-drought recovery in small waterbodies, and more generally in freshwater ecosystems, prevents any prediction of their structure and function in a context of drought intensification.

Experimentally assessing the ecological recovery after a drought in aquatic system is challenging because it requires the manipulation of water inputs at the entire ecosystem level, and with sufficient replication. To tackle these issues, we used tank bromeliads as a model ecosystem. Tank bromeliads are abundant terrestrial plants in Neotropical rainforests^26^. Their interlocking leaves form wells that collect rainwater (some milliliters to a few liters) and organic detritus (especially leaf litter). These small plant-formed waterbodies, or “phytotelmata”, harbor aquatic food webs that include microorganisms (bacteria, fungi and protists), and invertebrates from detritivores up to predators^27^. Their small size and strict physical delimitation allow for an exhaustive sampling of the aquatic communities and accurate characterization of their trophic structure. In particular, the ratio of predator to prey biomass in the invertebrate community has been shown to change along natural gradients in bromeliad size and climatic stability^28,29^. Consequently, tank bromeliads have proven to be useful for experimentally studying ecosystem processes and testing ecological hypotheses in nature^30–32^.

The main objective of this study was to understand how macroinvertebrate trophic structure and ecosystem function recover after droughts of different durations, from moderate to extreme droughts. We simulated dry periods by placing rain shelters above tank bromeliads. We monitored the resistance and recovery of invertebrate prey and predator biomass, as well as leaf litter decomposition, a key ecosystem function in this system, during the course of the post-drought, rewetting phase. In bromeliad ecosystems, food chains are short, with few predator species feeding on diverse prey populations mainly composed of detritivores. Thus, changes in predator and prey biomass, and the consequence on predator–prey biomass ratio, indicate changes in energy and nutrient fluxes within trophic pyramids^33^. Due to differences in population turnover rates and species’ energetic demands^34,35^, we predicted that predator and prey biomass would exhibit different recovery trajectories. The resulting asynchrony in predator and prey biomass recovery would impact key ecosystem functions driven by invertebrates. Because tank bromeliads are detrital-based systems, we hypothesized that changes in prey biomass (detritivores) during rewetting would be tightly linked to changes in leaf litter decomposition. We expected that asynchrony in predator and prey recovery trajectories will skew macroinvertebrate trophic structure and thus lengthen its post-drought recovery time, subsequently increasing the risk of exposure to a new drought prior to complete recovery. Overall, we expected that more frequent and longer droughts will hinder the recovery of small waterbodies structure and functions, ultimately weakening the resilience of these ecosystems in the face of environmental change.

## Results

### Prey and predator biomass

**Figure 1:**
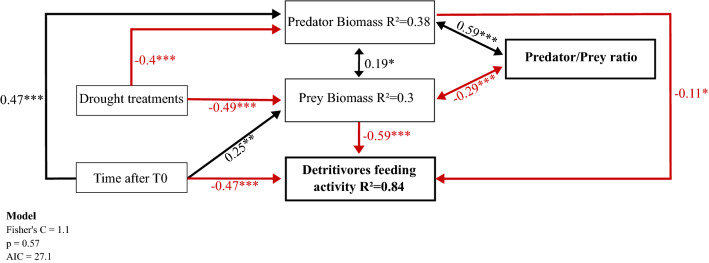
Structural equation model (SEM) of the relation between drought scenarios (drought treatment and time after T0), ecosystem structure (predator and prey biomass and their ratio) and function (detritivores feeding activity). Adjusted R-squared in the box indicates the percentage of variance explained by the model while numbers along the arrows indicate the weight of the path relationship. Black and red arrows respectively represent positive and negative significant relationships (* p-value < 0.05; ** p-value < 0.01; *** p-value < 0.001).

Longer drought treatments significantly reduced predator biomass (ANOVA: F_1:129_ = 33.3; p-value < 0.001; SEM path: − 0.4; p-value < 0.001) and prey biomass (ANOVA: F_1:129_ = 42.18; p-value < 0.001; SEM path: − 0.49; p-value < 0.001) (Fig. 1). During the rewetting phase, the elapsed time since drought ended (time after T0) significantly affected predator (ANOVA: F_1;129_ = 46.74; p-value < 0.001; SEM path: 0.47; p-value < 0.001) and prey (ANOVA: F_1;129_ = 10.83; p-value < 0.01; SEM path: 0.25; p-value < 0.01) biomass (Fig. 1). Drought treatment and time after T0 had no interactive effect on predator biomass (ANOVA: F_1;129_ = 2.38; p-value = 0.13) neither prey biomass (ANOVA: F_1;129_ = 2.0; p-value = 0.16). It is worth noting that time after T0 did not induce significant differences of prey biomass (ANOVA: F_1;12_ = 0.014; p-value > 0.05; SEM path p-value > 0.05), nor predator biomass (ANOVA: F_1;12_ = 4.35; p-value > 0.05; SEM path p-value > 0.05) in control bromeliads (Figure S2) suggesting that the aforementioned differences in drought treatments were the outcomes of the drought events and not of any external confounding factor.

### Predator–prey ratio

**Figure 2:**
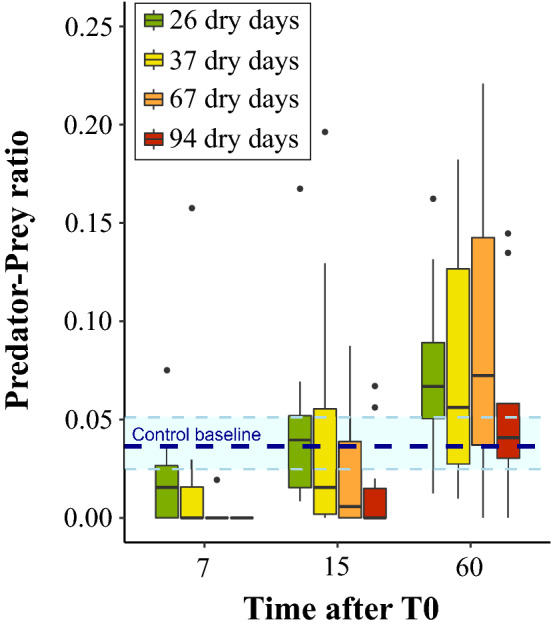
Predator–Prey biomass ratio versus time after rewetting (T0) and drought treatments. Higher values reflect an increasing proportion of predators versus prey. Boxes represent interquartile ranges with median values, and dots are outliers. The dashed dark blue line represents the control baseline and light blue range is the control interquartile range over the entire experiment for better graphical readability. Statistical tests were performed with control baseline individualized for each sampling period (T7, T15, T60).

Longer drought treatments significantly reduced the predator–prey biomass ratio (ANOVA: F_1;129_ = 12.43; p-value < 0.001). Time after T0 also significantly affected the predator–prey biomass ratio (ANOVA: F_1;129_ = 25.26, p-value < 0.001) which re-increased with elapsed time since drought ended (Fig. 2). Yet, no interactive effects of drought treatment and time after T0 were reported on the predator–prey biomass ratio (ANOVA: F_1;129_ = 2.2; p-value = 0.14). The dynamic of the recovery appeared slower after longer dry period. Seven days after T0, predator–prey biomass ratio was not significantly lower than control in the shorter drought treatment (26 dry days), a slight significant difference was reported for the intermediate drought treatment (37 dry days) and highly significant differences were reported for the two longest drought treatment (67 and 94 dry days). Fifteen days after T0 the trophic structure recovered in the three shorter drought treatment (26, 37 and 67 dry days) but remains significantly lower than control in the longest drought treatment (94 dry days) (Table 1). After 60 days of rewetting, the predator–prey biomass ratio in drought treatment bromeliads was no longer significantly lower from the control bromeliads (ANOVA: F_1;41_ = 0.95; p-value > 0.05) (Fig. 2, Table 1). Our SEM model revealed that predator–prey biomass ratio mainly depended on predator biomass (SEM path: 0.58; p-value < 0.001) rather than prey biomass (SEM path: − 0.29; p-value < 0.001) (Fig. 1). The prominent constraint of predator biomass on the recovery of the predator–prey biomass ratio results from a slower recovery of predator compared to that of prey during the rewetting phase (Figure S3). Finally, predator–prey biomass ratio in control bromeliads did not show significant differences during the “rewetting” phase (ANOVA: F_1;12_ = 1.88; p-value > 0.05), showing that differences reported were the outcomes of the drought events and not of any external confounding factor.

**Table 1:**
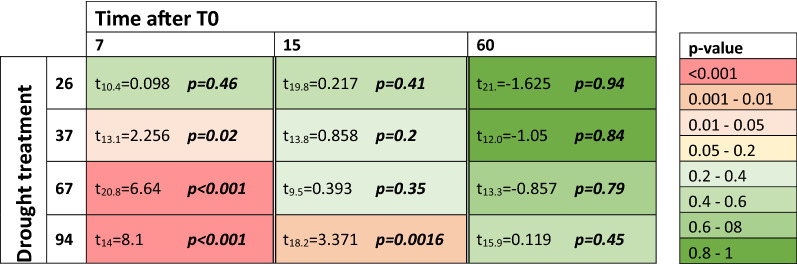
Dynamic of trophic structure recovery depending on drought treatment.

Values in each box are the result of a one-tailed T-test between sample and control predator–prey biomass ratio. Colors gradients reflects the level of significance of the “reduction versus control” relying on T-test p-values. The lower p-value is, the stronger is the reduction of predator–prey ratio in treatment versus control.

### Litter decomposition rate

Time after T0 exhibited significant, yet not surprising, effect on litter decomposition rate (ANOVA: F_1;125_ = 341.8; p-value < 0.001). Conversely, neither drought treatment (ANOVA: F_1;125_ = 0.02; p-value > 0.05) nor the interaction of the two factors (ANOVA: F_1;125_ = 2.2; p-value > 0.05) exhibited significant effect on litter decomposition rate. Litter decomposition rate thus remains similar regardless of the dry period undergone by bromeliads.

### Detritivores feeding activity (DFA)

**Figure 3:**
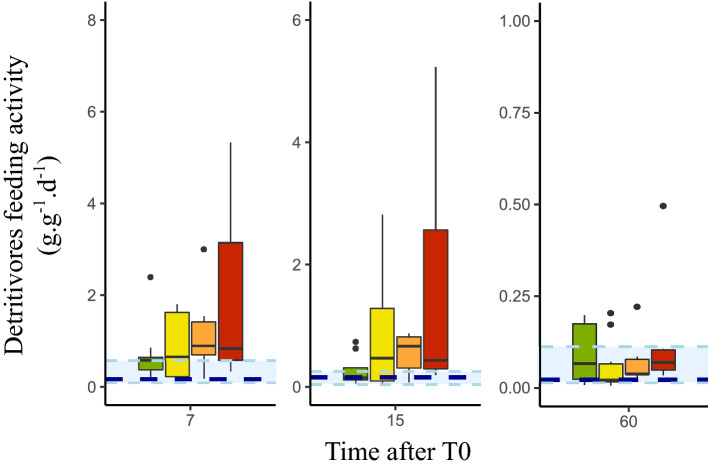
Detritivores feeding activity versus drought treatment at 7, 15 or 60 days after T0. Detritivores feeding activity is presented by gram of leaf litter mass loss per gram of invertebrate prey per day (g.g^−1^.d^−1^) the dashed dark blue line represents the control baseline and light blue range is the control interquartile range.

DFA was significantly affected by both time after T0 (ANOVA: F_1;125_ = 131.9; p-value < 0.001) and drought treatments (ANOVA: F_1;125_ = 28.6; p-value < 0.001) but not by their interaction (ANOVA: F_1;125_ = 1.17; p-value > 0.05) (Fig. 3). Our SEM model showed that DFA was negatively affected by the time after T0 both directly (SEM path: − 0.47; p-value < 0.001) and indirectly (i.e., mediated by prey biomass: SEM path: 0.25*–0.59 = − 0.15). Time after T0 has thus an overall strong and negative effect on DFA (total path: − 0.62). The effect of drought treatments on DFA was not direct (SEM path p-value > 0.05) but mediated by prey biomass (SEM path: − 0.49*–0.59 = 0.29) (Fig. 1). More specifically, drought treatments directly and negatively influenced prey biomass (path − 0.49; p-value < 0.001), the latter being negatively linked to DFA (path: − 0.59; p-value < 0.001). These sequential, negative relationships led to an overall positive effect of drought treatments on DFA. Drought also affected DFA positively, through its effects on predator biomass (path: − 0.4*–0.11 = 0.044).

## Discussion

Understanding the mechanisms that underpin post-drought recovery of communities is essential to accurately evaluate ecosystem response to climate changes and drought intensification. Here we showed that the recovery time of trophic structure, extrapolated from the predator–prey biomass ratio within a freshwater invertebrate community, increased with drought intensity in water-filled bromeliads. This occurred because the recovery of prey and predators was asynchronous. Before a full recovery was achieved, predator–prey biomass ratio remained low in all drought treatments, indicating alterations of the trophic structure of macroinvertebrate communities with potential consequences on litter decomposition. Thus, the recovery time of trophic structure could become critical to the maintenance of ecosystem function between successive drought events.

The immediate effect of drought on ecosystems is modulated by the direct resistance of communities, which depended on the desiccation tolerance of invertebrate species of our study area^20^. Here, a decrease of the predator–prey biomass ratio with increasing drought intensity revealed a higher sensitivity to drought of predator than prey assemblages. This higher sensitivity can be partly explained by the larger body size and higher energetic demands of predators, that make them more sensitive to shrinking water volumes in bromeliads. This is also consistent with predictions of the trophic rank hypothesis arguing that the effects of environmental constraints multiply as the trophic level increases because of trophic dependencies between predators and prey^36,37^. Yet, this high sensitivity of predators to drought should be nuanced. In some contexts, by reducing water volume without affecting predator survival, dry periods may concentrate prey, facilitate the feeding of predators and thus improve their productivity^32^. Here, although higher predation rates likely occurred as water volume declined^38^, they were insufficient to compensate for the direct loss of predator biomass that led to a reduction of the predator–prey ratio at the beginning of the rewetting phase (T7). The fragile balance between persistence or extinction of predators depending on the duration of drought and abundance of their prey suggests that stochastic extinction risk can be modulated by coexistence with other populations and/or species^39^. In our case, stochastic extinction of predators, especially when drought extended over long periods, may have important consequences on community structure that may reverberate on their overall resilience. While this specific point is beyond the scope of the present study, as we did not focus on the processes occurring during the dry phase, its potential importance should encourage further works.

From its low level, the predator–prey ratio increased up to control values during the rewetting phase, reflecting the recovery of ecosystem trophic structure after a drought event. The gradual recovery of the predator–prey ratio was the result of the slower recovery of predator biomass relative to prey biomass. Indeed, prey biomass reached high values early in the rewetting phase due to their higher resistance to drought and/or faster recovery after the dry period. Conversely, the recovery of predator biomass lagged behind that of prey. This lag can be attributed to two processes. First, the strong effect of drought reducing predator biomass to near zero after most drought treatments (37, 67 and 94 dry days) resulted in predator recovery initiating from a lower level. Second, the predator biomass is limited by the availability of prey biomass and inevitably increased with a time lag in a similar pattern to what was reported by the well-known Lotka-Volterra model^40^. This delayed and gradual recovery of predator biomass relative to prey explains the increase in the predator–prey ratio observed between 7 and 60 days of rewetting. Although the general tendency was comparable regardless of the drought treatments, recovery time appeared to be mediated by the duration of the dry phase. Organisms recovered within 7 to 15 days after short dry periods (26 days), indicating that resident communities were fairly adapted to such events, which naturally occurred for several decades^23^. However, when drought extended over longer periods (94 dry days), recovery time reached up to 60 days. While these results revealed that longer dry periods lengthen ecosystem recovery time, the absence of significant interaction between drought treatments and time after T0 suggests that these differences rely on the starting point of recovery rather than on differences in the recovery rate. In other words, the longer recovery time is not a direct result of differences in recovery rate but depends mainly on community biomass at incipient rewetting. This could have consequences if an additional drought was to occur within the recovery period, as it could reduce the “starting point” to a lower level than expected. Even short, but repeated dry periods, could ultimately reduce invertebrate biomass down to critical levels, eroding trophic structure more than a single long dry period. Interestingly, this conclusion can be transposed to the “press and pulse” framework used to describe ecological disturbances^41,42^. Each drought event can be seen as pulse disturbance in that it constitutes a temporary event constraining ecosystem structure and functions. In our case, pulse disturbance had severe, yet reversible consequences for ecosystem trophic structure. On the other hand, the increased frequency of droughts can be seen as a press disturbance affecting bromeliad ecosystems at longer timescales. Press and pulse disturbances are known to interactively affect population dynamics and persistence^41,43^, suggesting that drought events would have stronger effect if they were to become more frequent. A related issue is that the state of the community or ecosystem when a drought event occurs will have important implications on its subsequent resilience.

Besides altering ecosystem structure, droughts may also affect ecosystem functions such as litter decomposition. After dry periods, the reduction of prey biomass, mainly constituted of detritivores organisms, was expected to lead to a reduction of the overall litter decomposition rate. Yet, our result revealed that droughts did not affect litter decomposition rate which remains similar regardless of our drought treatments because of compensatory processes existing between prey biomass and feeding activity. First, it appeared that interindividual competition^44^ led to the negative relation between prey biomass and individual feeding activity reported in our model. Thus, the drought-induced reduction of detritivores biomass may have alleviated competition for food and/or space^44^, allowing higher individual feeding rates. Second, our model revealed that DFA is negatively affected by predators biomass, a relation arising because higher level of activity often increase the predation risk^45^ leading prey to be less active in presence of predators. Yet in post-drought conditions, the absence of predators reduced the risk of predation and subsequently allowed an increase of prey (i.e. detritivores) feeding activity. A third, more hypothetical, explanation for an increased DFA could relate to drought-induced differences in the quality of detrital resources. By inducing animal mortality, droughts could lead to an accumulation of dead organisms. Such organic matter rich in protein compared to C-rich dead leaves, could form a source of nutrient for detritivores^46^, increasing their overall performance and feeding capacities. Overall, the simultaneously drought-induced reduction of detritivores biomass and increase of their individual feeding activity compensate each other leading to the reported stability of the overall litter decomposition rate.

Water availability and precipitations regimes are known as major factors controlling litter decomposition in Neotropical forests^47^. Under climatic scenarios where drought became more frequent and severe, decomposition is expected to be affected by both physiochemical and biological processes^48,49^. The alternance of dry and wet periods tends to leach and fragment leaf litter facilitating its decomposition. Conversely, by altering microbial communities and the associated microbial decomposition^31^^,^^49^ longer droughts are expected to reduce the overall decomposition rate. Here we showed that DFA significantly increased after long drought periods. We suggest that such increase of invertebrate activity may partly compensate for the drought-induced loss of microbial decomposition. Moreover, the fragmentation of leaf resulting from shredder activity may also facilitate the microbial post-drought recolonization, ultimately compensating the loss of microbial decomposition induced by long droughts. Overall, invertebrate decomposition, while often being considered of minor importance for leaf litter decomposition in Neotropical forest under normal hydrological conditions^31,49^, may become critical in a context of drought intensification. These results are consistent with those reported in terrestrial environments where an increased importance of invertebrate in litter decomposition was observed after severe perturbations^50^.

We conclude that asynchrony in the post-drought recovery time of predator and prey invertebrate assemblages is a critical driver of the recovery trajectory of trophic structure and ecosystem function. Though we took a functional approach to community resilience to drought, unraveling the underpinning life-history traits could further enhance our understanding of ecosystem resilience to drought. Information on population dynamics could notably explain patterns of species turnover during the recovery phase. Still, our results provide evidence that the higher sensitivity and longer recovery time of predators determine the recovery kinetics of ecosystem trophic structure after drought. Longer recovery increases the risk of another drought before returning to a pre-disturbance stage, thus weakening the resilience of small waterbodies to repeated drought events. Such events will further damage the functional and long-term integrity of small water bodies in the context of global change where precipitations are expected to reduce by up to 50% over the twenty-first century (IPCC scenario RCP 8.5). We showed that release of top-down control by predators makes detritivores more active during the post-drought period. This led to a more rapid recovery of their biomass and undoubtedly to an increase in their feeding activity allowing to keep a stable level of leaf litter decomposition. Ecosystem resilience to drought thus depends on a fragile balance between predator versus prey resistance, and recovery capacities, which determine the maintenance of trophic structure on one hand and the recovery of ecosystem functions on the other. The primary role of predators in maintaining this equilibrium coupled with their high sensitivity to environmental stressors should encourage further studies to specifically explore the direct and/or indirect role of higher trophic levels for ecosystem resilience to drought but also other environmental alterations associated to global change.

## Method

### Study site

The study site was in a primary rainforest understory situated in French Guiana near the Petit-Saut hydroelectric dam (5° 03′ 43ʺ N, 53° 02′ 46ʺ W). In this tropical wet environment, temperatures exhibit low seasonal variation with monthly mean temperatures ranging from 20.5 to 33.5 °C. The relative humidity ranges between 70 and 100%, with 3000 mm precipitation per year. The rainy season covers 280 days a year and is only interrupted by a long dry season from September to November and a shorter and less regular one in March.

### Bromeliad selection

Our experiment was conducted with *Lutheria splendens* (Bromeliaceae), the only tank bromeliad species at the study site^35^. Taxonomic identification of *Lutheria splendens* was performed by C. Leroy with vouchers referenced with the Cayenne Herbarium (CAY). Permission to collect plant material was obtained under the Internationally recognized certificate of compliance ABSCH-IRCC-FR-247-227-1 issued by the French Ministry of Ecology. In accordance with the above-mentioned permit, manipulation and collection of plant material in this study complied with relevant national and international guidelines and legislation. Briefly, we selected 135 mature *L. splendens* with comparable vegetative traits (number of leaves, plant diameter) evenly distributed within a 1 ha forest plot.

### Drought treatment selection and simulation

**Figure 4:**
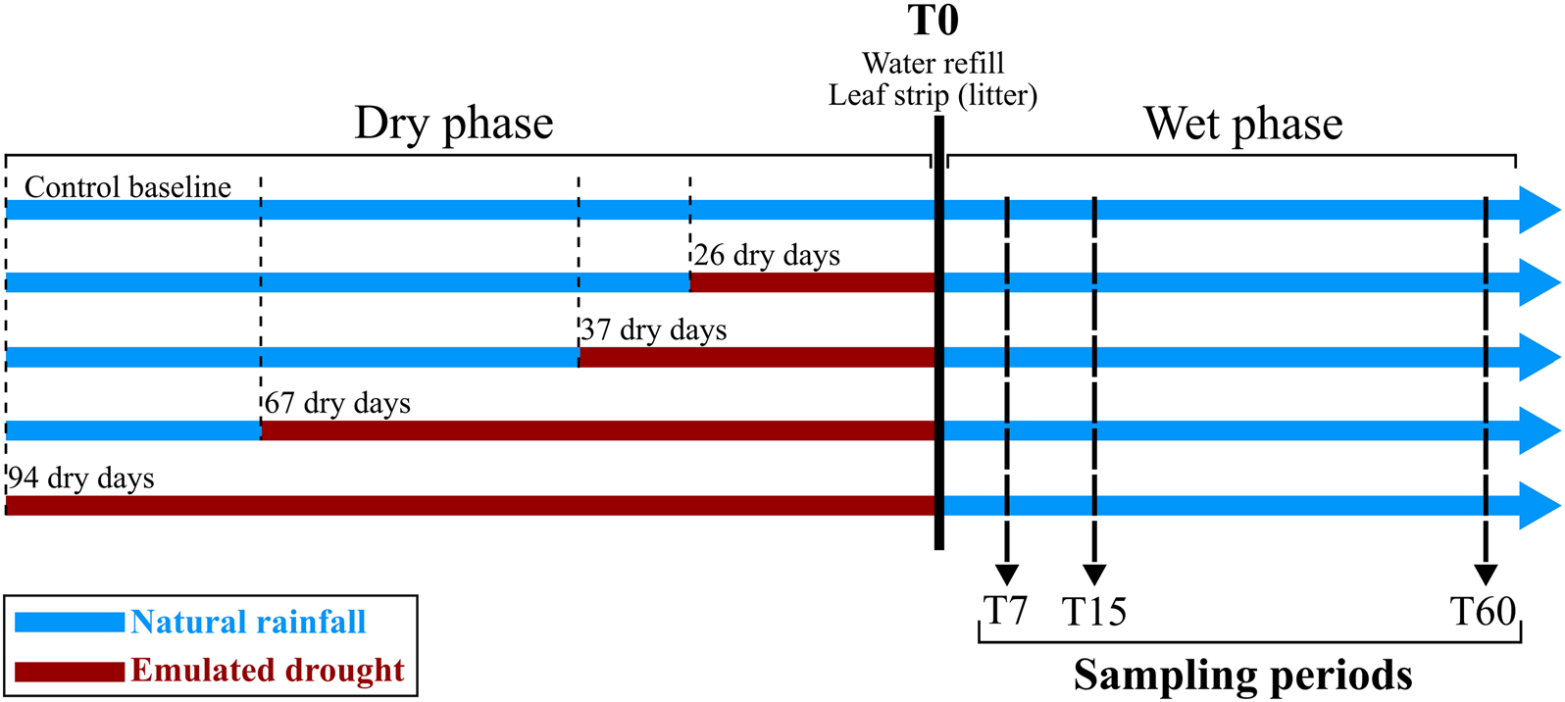
Experimental timeline of the four treatments emulating different drought durations (dry phase, before T0) and sampling periods (wet phase, after T0). The control baseline undergone natural rainfall all along the experiment duration.

Bromeliads were divided into 4 groups of 30 individuals each (n = 120) submitted to four different drought conditions (see below), plus one control group (n = 15) undergoing natural rainfall. The drought treatments used as a reference represents the mean number of consecutive days without rainfall (26 days ± SD 5.3) and the most extreme event (67 days) observed over the last 20 years (daily rainfall recorded at the Paracou, French Guiana weather station). We then coupled this historical observation with the IPCC forecast of a decrease of precipitation between 10 and 50% in north Amazonia during the twenty-first century. Thus, our treatments consisted of 26, 37, 67 and 94 consecutive days without rainfall, which represent, respectively, the current mean number of dry days in the area, the mean + 40%, the most extreme drought ever recorded over the past 20 years, and the extreme + 40%. To simulate these drought events, rain shelters made of transparent tarpaulin were placed 1 m above each plant to prevent natural supply of rain. These rain shelters have been successfully used in many experiments with bromeliads and do not affect incident light nor ambient temperature^51^. The sets of rain shelters simulating different drought intensities were staggered in time, so the various drought treatments ended simultaneously (Fig. 4). With these settings, the shortest 26-day drought treatment matched the irregular dry season, and the 37–67–94 days treatments emulated longer droughts over periods that would have otherwise been rainy. The pre-drought conditions for all bromeliads were typical of a rainy season, where bromeliads at our site are generally filled at nearly 50% of their capacity (see survey^34^). Thus, before the rewetting phase, bromeliads essentially differed by the dry period they experienced. At the end of the dry phase (hereafter T0), tank bromeliads were refilled to their capacity with rainwater, and rain shelters were removed. Bromeliads then underwent natural rainfall during the two months of the “wet phase”. For full transparency we report that half of the treatment bromeliads were covered with mosquito nets during the rewetting phase, so the role of immigration on community dynamics could be evaluated in a separate experiment^15^. The results unambiguously showed that the post-drought community dynamic was entirely supported by in situ resistance, not immigration. We further verified that netting on bromeliads had no significant effect on our results (see Table S1). Hence, net presence or absence is not considered further in the present study and all bromeliads were used in our data analysis.

### Leaf strips decomposition

One of our objectives was to assess leaf litter decomposition directly associated to invertebrate. To do so, freshly fallen leaves of *Goupia glabra* (Goupiaceae) were collected in litter traps, hydrated in filtered rainwater for 24 h, and then cut into strips. As reported above, plant collection was allowed under Internationally recognized certificate of compliance ABSCH-IRCC-FR-247-227-1 and followed relevant guidelines. Identification was performed by C.Leroy with vouchers referenced with the Cayenne Herbarium (CAY). *G. glabra* is a very common tree in this part of French Guiana whose leaves are frequently found in tank bromeliads. This taxon has among the most rapidly decomposing leaf litter in French Guiana soils^52^ and has already been used effectively to follow leaf litter decomposition in tank bromeliads^31^. Before incubation, *G. glabra* leaf strips were oven dried at 60 °C for 48 h and weighed to the nearest 0.1 mg. This drying drastically reduces the microbial communities in the leaves. At T0, each individual plant received two 2 × 4 cm leaf strips of *G. glabra* in two wells with the same water holding capacity, in order to follow the decomposition (i.e. leaf mass loss rate) during the rewetting phase. The introduction of leaf strips at T0 allows the exclusion of drought-induced leaf fragmentation which is known to affect leaf decomposition^49^. Moreover, the absence of difference in microbial colonization between drought treatments suggests that microbial decomposition should be similar. For these reasons, we assumed that between-treatment differences in litter decomposition rate mainly resulted from differences in detritivore feeding activities.

### Sampling design and sample processing

During the rewetting phase, we individually sampled the macroinvertebrates and removed the leaf strips from 10 bromeliads per drought treatment and 5 control bromeliads at 7, 15 and 60 days after T0. Thus, in total, our experimental design consisted of 4 drought treatment × 3 rewetting periods (n = 10, N = 120) plus a single control treatment at 3 rewetting periods (n = 5, N = 15). Aquatic invertebrates were sampled using 10 mL micropipettes with the end trimmed to widen the aperture. The entire water content of bromeliads was pipetted carefully to ensure the most complete sample of aquatic invertebrates (larva and/or adults). Invertebrates were sorted by species or morphospecies and enumerated. Species’ biomass were estimated with allometric relationships between body length and dry mass^20,34^. We sorted invertebrate taxa into two trophic levels: prey or predator^53^ (Table 2). Leaf strips sampled from each bromeliad were oven dried at 60 °C for 48 h and weighed to estimate the gross decomposition rate since T0.

**Table 2:** Invertebrate taxa sampled in our experiment.

Species/Morphospecies	Feeding group	Trophic level
*Anophele nevai*	Filter feeder	Prey
*Aulophorus superterrenus*	Deposit feeder	Prey
*Bezzia*	Predator	Predator
*Cecidomyiidae*	Piercer	Prey
*Chironomini*	Filter feeder	Prey
*Copelatus sp.*	Predator	Predator
*Corethrella sp.*	Predator	Predator
*Culex sp.*	Filter feeder	Prey
*Cyphon sp.*	Scraper	Prey
*Elmidae*	Scraper	Prey
*Elpidium bromeliarum*	Deposit feeder	Prey
*Enchytraeidae*	Deposit feeder	Prey
*Microculex stonei*	Filter feeder	Prey
*Microstigma maculatum*	Predator	Predator
*Orthocladinae*	Deposit feeder	Prey
*Ostracoda unk*	Deposit feeder	Prey
*Paravelia recens*	Piercer / Predator	Predator
*Scrites sp.*	Scraper	Prey
*Spheridinae*	Piercer	Prey
*Trentepohlia sp.1*	Shredder	Prey
*Trentepohlia sp.2*	Shredder	Prey
*Turbellaria*	Predator	Predator
*Wyeomyia aphobema*	Filter feeder	Prey

Each species/morphospecies are associated to their feeding group^53^ and trophic level.

### Data analysis

Prior to statistical analysis, the predator biomass, prey biomass and predator–prey biomass ratio were square root transformed to normalize data dealing with 0 values. Leaf litter decomposition, calculated as litter mass loss (g), was divided by total detritivores biomass (g) and time (days). Leaf litter decomposition per unit of invertebrate detritivores biomass per unit of time (g.g^−1^d^−1^) is thus a proxy of detritivore feeding activity (thereafter DFA) at the level of detritivores community. Leaf decomposition rate and detritivores feeding activity were log transformed to meet model assumptions.

We used piecewise structural equation model (SEM) to define the direct and/or indirect effects of our experimental factors (drought treatment and time after T0) on our measured variables (prey and predator biomass, predator/prey ratio, DFA). Using a priori knowledge, we defined hypothetical relationships within a path diagram (Figure S1, Table S2) from which model was constructed. Model fit was tested using Fisher’s statistic and Akaike Information Criterion (AIC). Starting from the output of our a priori model and step-by-step exclusion and selection of variables, we found the model with the lowest AIC value. We ran the SEM analysis using the R package *piecewiseSEM*^54^ providing the effect size and significance level of each path (a.k.a. causal link) of the model. To test whether the differences reported in our model derived from the treatments rather than from other confounding factors, we ran the model on the datapoints of control bromeliads only and compare the path significance and effect size with the model displaying all datapoints.

In addition, we tested the total effect of drought treatments and time after T0 on predator biomass, prey biomass and predator–prey biomass ratio using ANOVA. In a comparable way than reported for SEM model, we tested the effects of time after T0 on predator biomass, prey biomass and predator–prey biomass ratio on control treatment only to test whether confounding factors may have affected our results. To define recovery dynamics of ecosystem structure, we compare the predator–prey biomass ratio in each drought treatment and each time after T0 versus control using student one-tailed T-test. The strength of the reduction of the predator–prey ratio between control and samples was based on T-test p-value and its evolution at different times after T0 was used to estimate the recovery dynamic in each drought treatment.

Finally, we tested the effect of drought treatments and time after T0 on litter decomposition rate using ANOVA.

## Supplementary Information

Supplementary Information.

## Data Availability

The data of the manuscript is available on Figshare at 10. 6084/m9.figshare.16794742.
