# Supplementary material for: Asynchronous recovery of predators and prey conditions resilience to drought in a neotropical ecosystem
Source: Sci Rep. 2022 May 19;12:8392. doi: 10.1038/s41598-022-12537-2 (PMC9120075; doi:10.1038/s41598-022-12537-2)
Supplement: Supplementary file 1 — Supplementary Information. [file 41598_2022_12537_MOESM1_ESM.pdf]

Supplementary Materials for :

Asynchronous recovery of predators and prey conditions resilience to drought in a Neotropical ecosystem

Thomas Ruiz, Jean-François Carrias, Camille Bonhomme, Vinicius F. Farjalla, Vincent E.J. Jassey, Joséphine Leflaive, Arthur Compin, Céline Leroy, Bruno Corbara, Diane S. Srivastava, Régis Céréghino

	df	Sum sq	Mean Sq	F-value	Pr(>F)
Drought.treatment	1	0.2717	0.2717	12.478	0.000572 ***
Time.after.T0	1	0.5517	0.5517	25.341	1.58E-06 ***
Colonization.treatment	1	0.0572	0.0572	2.629	0.107371
Residuals	129	2.805	0.0218		

Table S1: Statistical results of a model exploring the response of predator-prey ratio to presence of net above bromeliads (colonization.treatment). Comparison was performed using an ANOVA analysis where predator-prey ratio was tested versus drought treatemnts, time after T0 and colonization (cover with net or not). It appears that netting has no significant effects on the differences of predator-prey biomass ratio reported in this study.

Path	Causal hypothesis
D.Pred	Drought duration affects predator biomass (resistance)
D.Prey	Drought duration affects prey biomass (resistance)
T.Pred	Time after T0 affects predator biomass (recovery)
T.Prey	Time after T0 affect prey biomass (recovery)
Prey.Pred	Predator biomass requires resources (prey) to re-grow after drought
Pred.R	Predator biomass constraints predator-prey ratio
Prey.Ratio	Prey biomass constraints predator-prey ratio
D.DFA	Drought treatment may affect detritivores feeding activity du to direct stress (resistance)
T.DFA	Time after T0 may affect detritivores feeding activity (recovery)
Prey.DFA	Prey biomass may affect interindividual competition for food with consequences on their feeding activity

Table S2: Hypothesis underlying the path of the structural equation model.

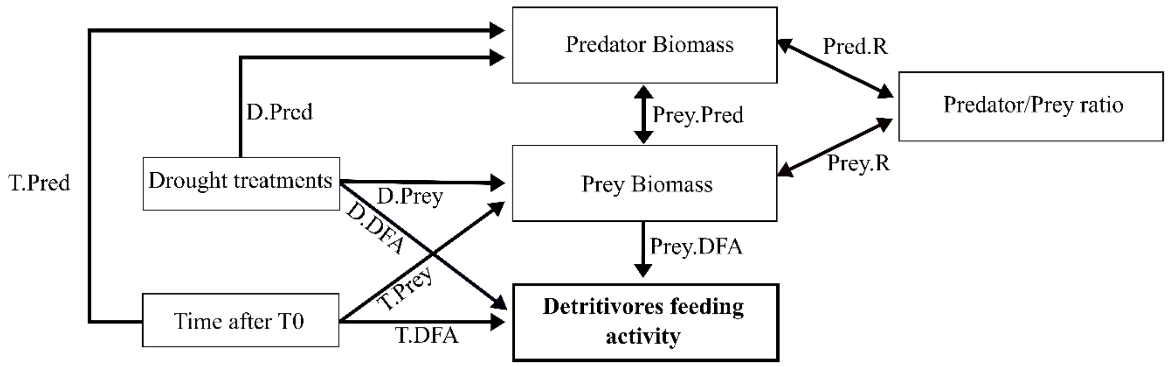

<u>Figure S1:</u> A priori conceptual structural equation model (SEM) depicting pathways by which predator and prey biomass may affect the specific decomposition rate. Letters correspond to hypothetical pathways (Table S2).

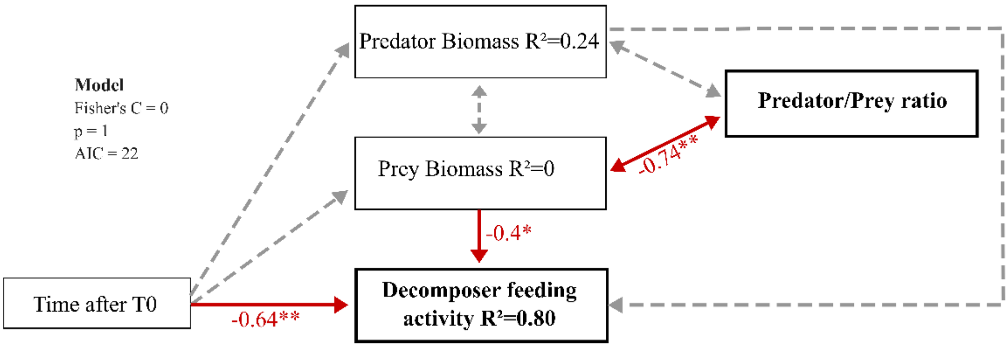

<u>**Figure S2:**</u> **Structural equation model (SEM) linking time after T0, ecosystem structure (predator and prey biomass) and function (detritivores feeding activity) on the control bromeliads only.** Adjusted R-squared in the box indicate the percentage of variance explained by the model while numbers along arrow indicate the weight of the path relationship. Black and red arrows respectively represent positive and negative significant relationships, grey dashed arrows reflect unsignificant relations (* p-value<0.05; ** p-value<0.01; *** p-value<0.001).

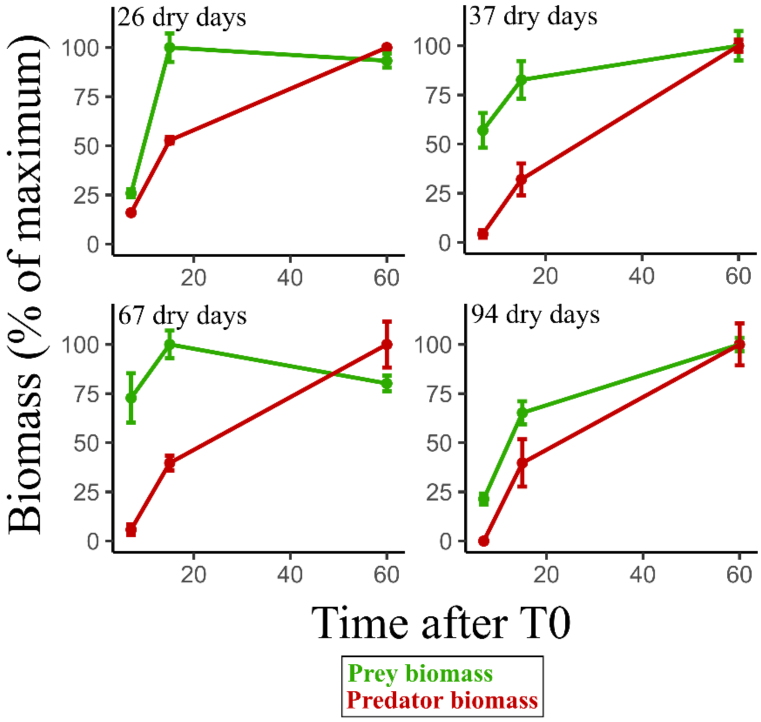

<u>Figure S3:</u> Model of Prey and Predator biomass dynamic in post-drought conditions. Each panel refers to a different drought treatment (26, 37, 67 or 94 dry days). Biomass of each trophic level is given in percentage of maximum reported for each drought treatment individually. Error bars represents 95% confidence intervals generated by non-parametric bootstrapping. Recovery of predator biomass systematically occurs after the recovery of prey biomass.
